# How and to what extent can pensions facilitate increased use of health services by older people: evidence from social pension expansion in rural China

**DOI:** 10.1186/s12913-020-05831-0

**Published:** 2020-11-04

**Authors:** Shanquan Chen, Xi Chen, Stephen Law, Henry Lucas, Shenlan Tang, Qian Long, Lei Xue, Zheng Wang

**Affiliations:** 1 The School of Clinical Medicine, University of Cambridge, Cambridge, UK; 2 Department of Health Policy and Management, Yale School of Public Health, New Haven, CT USA; 3 Faculty of Medicine and Health, University of Sydney, Sydney, Australia; 4 Institute of Development Studies, University of Sussex, Brighton, UK; 5 Department of Population Health Science, Duke Global Health Institute, Duke University, Durham, North Carolina USA; 6 Global Health Research Center, Duke Kunshan University, Kunshan, Jiangsu China; 7 Institute for Hospital Management, Tsinghua University, Beijing, China; 8 Key Laboratory of Environmental Pollution Monitoring and Disease Control, Ministry of Education, Guizhou Medical University, Guiyang, 550025 China; 9 Research Center for Healthcare Management, School of Economic and Management, Tsinghua University, Beijing, 100084 China

**Keywords:** Pension, Health service utilization, Regression discontinuity design, Segmented regression

## Abstract

**Background:**

The proportion of people aged 60 years or over is growing faster than other age groups. Traditionally, retirement has been considered as both a loss to the labour market and an additional economic burden on the nation. More recently, it is widely accepted that retired people can still contribute to society in many ways, though the extent of their contributions will depend heavily on their state of health. In this context, a significant practical issue is how to encourage older people to use the health services they need. This study aims to evaluate the effects of pensions on older adults’ health service utilization, and estimate the level of pension required to influence such utilization.

**Methods:**

Using data from a nationally representative sample survey, the China Health and Retirement Longitudinal Study, we adopted a fuzzy regression discontinuity design and undertook segmented regression analysis.

**Results:**

It was found that a pension did encourage low-income people to use both outpatient (OR = 1.219, 95% 1.018–1.460) and inpatient services (OR = 1.269, 95% 1.020–1.579); but also encouraged both low- and high-income people to choose self-treatment, specifically over-the-counter (OR = 1.208, 95% 1.037–1.407; OR = 1.206, 95% 1.024–1.419; respectively) and traditional Chinese medicines (OR = 1.452, 95% 1.094–1.932; OR = 1.456, 95% 1.079–1.955; respectively). However, receiving a pension had no effect on the frequency of outpatient and inpatient service use. Breakpoints for a pension to promote health service utilization were mainly located in the range 55–95 CNY (7.1–12.3 EUR or 8.0–13.8 USD).

**Conclusions:**

A pension was found to have mixed effects on health service utilization for different income groups. Our study enriches existing evidence on the impact of pensions on healthcare-seeking behaviour and can be helpful in policy design and the formulation of improved models relating to pensions and healthcare utilisation.

**Supplementary Information:**

The online version contains supplementary material available at 10.1186/s12913-020-05831-0.

## Background

In almost every country, the proportion of people aged 60 years or over is growing faster than other age groups [1]. Globally, the number of persons in this age group is projected to grow by 56% by 2030 [2], and 80% will be living in low-and middle-income countries by 2050 [2, 3]. Traditionally, retirement has been considered as both a loss to the labour market and an additional economic burden on the nation. More recently, it is widely accepted that retired people can still contribute to society in many ways, though the extent of their contributions will depend heavily on their state of health [4]. Therefore, the relationship between retirement and health status has become an important topic of practical significance.

The effects of retirement on health have been extensively investigated. Many studies have reported unexpected changes in health status around the age of retirement [5] and indicated that retirees’ health status will often decline after involuntary retirement but improve if they chose to retire [6]. Several theories attempt to conceptualize the underlying mechanism by which retirement affects health. Jahoda’s latent deprivation theory [7, 8] proposes that job loss deprives retirees’ manifest functions (financial rewards) and latent functions (time structure, social contact, collective purposes, social identity or status, and regular activities), and thus heighten their distress level. Elder’s life course perspective [9] suggests that retirement affects individuals’ health by influencing their social relationships with family, friends, and co-workers. Grossman’s health capital model [10] observes that retirement increases an individual’s leisure time and decreases the opportunity costs of certain health investments. Some empirical studies also provided possible pathways by which retirement may affect health. For instance, Insler [11] reports a reduction in smoking and an increase in physical activity among retirees. Eibich [12] notes a relief from work-related stress and strain, an increase in sleep duration, and an increase in physical activity. Significantly, Rhee and colleagues [6] explore three theoretically plausible mechanisms based on previous evidence: financial control, family relationships, and social integration, and conclude that financial control was the dominant factor linking retirement and well-being.

Simultaneously, another area of research directly explores the relationship between retirees’ economic insecurity and their health status, focusing on the effects of conditional cash transfers (CCTs), for example, health vouchers in Hong Kong [13] and some states in the United States [14, 15], or unconditional cash transfers (UCTs), such as pensions in Brazil [16], Colombia [17], Mexico [18], and South Africa [19]. A recent review conducted by Pega [20] finds ample evidence that CCTs promotes retirees’ health status, while evidence on UCTs is more limited and inconsistent. Even so, UCTs are preferred by a number of researchers as being able to generate greater behaviour change, given that they are more socially acceptable and less stigmatizing for recipients than CCTs [20]. In addition, existing evidence suggests that universal expansion of health insurance and services might not be sufficient to improve the health of the whole population, because the costs of transport, subsistence and co-payments will still impede access to services, especially for the poor [17, 21, 22]. The flexibility of UCT may offset these barriers. In addition, the rapid growth in the number of older people and low coverage of social security reinforce the need for a comprehensive social protection system [18], perhaps most simply achieved using a UCT approach.

Health status is a long-term outcome of a complex process. Even if a study identifies an unchanged health status at specified times before and after a UCT, it cannot infer that the UCT has made no positive contribution. Therefore, researchers have also focused on the impact of UCTs on health service utilization, and suggested that evidence from such studies can be helpful in understanding the behaviour of retirees and designing suitable policies [5]. However, limited studies have addressed this issue to date [5, 20], with inconsistent results at the aggregate level. These include a negative effect in Europe [23], positive effects in 10 European countries [5] and Brazil, negligible effects in Germany [12] and the United States [12, 24–26], and a mixed effect in Colombia [17]. Given that the poor are more sensitive to the costs of transport, subsistence and co-payments [22], and thus may benefit more from a UCT, analysis by income group should offer more helpful insights. In this study, we aim to fill this gap in the literature using longitudinal national survey data.

As emphasized by Coe [27], the aim should be to look for the causal effect of UCT on health service utilization, not simply correlation between these variables. However, as illustrated above, other factors besides financial status, for example increased leisure time, not only accompany retirement but also influence the healthcare seeking behaviour of retirees. The potential confounding effects of such variables needs to be taken into account. In addition, access to a pension is typically associated with the near simultaneous loss of regular employment or self-employment income, and it is therefore the net effects on financial status that must be taken into account. A randomized controlled trial (RCT) study design might be used to address these issues but practical and ethical issues make such an approach unrealistic.

The above challenge will be addressed in two ways in this study. First, the study will focus on elderly rural residents. The New Rural Old Age Insurance (NROAI), a UCT program, was piloted in China in 10% of rural areas in late 2009 and then was promoted nationwide [28]. All residents aged 60 in the rural area covered by the NROAl will have the right to receive a retirement pension. The minimum basic pension for each participant is 55 Chinese Yuan (CNY) (7.12 EUR or 7.98 USD) per month, fully subsided by the government. For this rural population, because their income is primarily from agriculture, reaching the official retirement age will typically not greatly influence their agricultural and other economic and social activities [29, 30]. There is usually no loss of employment or self-employment income or direct impact on their leisure time. Second, a regression discontinuity design will be adopted to estimate the causal relationship as described below.

The study will examine the effect of the NROAI pension on outpatient health service utilization, inpatient utilization, and self-treatment across different income groups. The overall hypothesis is that the offered pension will facilitate elderly people, especially the poor, in greater utilization of health services. To provide additional evidence, and to offer practical suggestions as to how similar pension policies might be implemented in other settings, especially low- and middle-income countries (LMIC), we will also attempt to estimate the level of pension required to encourage increased health service utilization by recipients.

The paper is divided as follows: section 2 presents the methodology, including the data and variables used in the paper, indicators used to measure health service utilization, the regression discontinuity model and segmented regressions; section 3 presents the results, including the main analysis, validation and robust tests; and section 4 provides a discussion and conclusions.

## Methods

### Data source

The data used in this research were obtained from a nationally representative sample survey, the China Health and Retirement Longitudinal Study (CHARLS) for 2011, 2013 and 2015. Detailed descriptions of this data, sampling method and quality-control procedures have been reported elsewhere [31]. This household survey is conducted biennially and covers 450 villages/urban communities in 126 counties/districts located in 28 provinces across the country. A multistage, stratified random sample of people aged 45 years and over was collected in each wave of the survey.

Using a fuzzy regression discontinuity design (described below), a sub-sample of 14,922 CHARLS participants was selected based on the following inclusion and exclusion criteria:

- the participant was a rural resident, aged 50–70 (near the cut-off 60 with bandwidth 10);
- the participant was not covered by other pension schemes;
- to exclude interaction with medical insurance, the participant was enrolled in the new rural cooperative medical system (NCMS), a national social health insurance covering almost 95% of rural residents;
- to exclude the influence of retirement on income, the participant was not engaged in economic activity influenced by reaching the age of 60; this criterion restricted the analysis to those self-employed or participating in a family business.

### Dependent variable

In terms of health service utilization, previous studies have usually focused on facility-based outpatient and inpatient services. Most describe the process of visiting a doctor as composed of two steps [32–36]. The first involves a *contact decision*, i.e. the patient deciding to contact a physician. The second is designated a *frequency decision*, pertaining to repeated visits or referrals. An intervention may impact on either or both steps and we therefore consider both in this study. The relevant survey questions are:

- Outpatient contact decision: In the last month have you visited a public hospital, private hospital, public health centre, clinic, or consulted a doctor or other health worker, or been visited by a doctor or other health worker to provide outpatient care?
- Outpatient frequency decision: How many times did you visit or have you been visited during the last month?
- Inpatient contact decision: Have you been admitted to hospital in the past year?
- Inpatient frequency decision: How many times have you been admitted during the past year?

We will also consider self-treatment because of its high prevalence. For example, in another national survey in China, 27% of respondents reported self-treatment in 2008 [37]. In addition, due to the lower cost, a retirement pension may encourage retirees to try self-treatment before seeking care from a qualified provider. The definition of self-treatment varies [38–41]. According to the World Health Organization (WHO) [42], it is defined as “the activities that individuals, families, and communities undertake, with the intention of enhancing health, preventing illness, limiting illness, and restoring health.” Following this definition, we include the following actions undertaken in the absence of a consultation with a qualified health worker (1): consumption of over-the-counter modern medicines (2); consumption of traditional herbs or medicines (3); consumption of tonics or health supplements (4); using health care equipment.

### Covariate variables

The Andersen health behaviour model is the most common framework used in the study of health service utilization [43–47]. It was used to identify covariates needing to be included in the analysis. This model suggests that health service utilization for an individual is a function of a predisposition to use health services, factors that enable or impede such use, and the need for care [43, 47]. In this study, gender, education level, and living alone were considered as predisposing factors [48–51]. The single enabling factor included was household disposable income per capita per year [48–50]. The need factors included: Activities of Daily Living (ADL) index, self-rated health status, whether the patient reported body pains and whether they had been diagnosed with a chronic condition [48–50, 52, 53]. As our data come from years of survey, the survey year was also controlled as a covariate. Additional file 1: Appendix 1 of the supplementary materials gives more details of the definition and measurement of the covariate variables. Employment and pension incomes were specified in CNY but also presented in other currencies using the average exchange rate in 2019 (1 CNY = 0.12946 EUR, 0.145 USD).

### Analysis one: regression discontinuity

The regression discontinuity (RD) design is a rigorous quasi-experimental approach that can be used to estimate intervention impacts as long as the intervention adopts a continuous measure (force variable) with a clearly defined threshold (cut-off score) to determine who is eligible and who is not [54]. RD can both identify causal relationships and mitigate the endogenous problems arising from reverse causality and misspecification [55–58].

The idea of RD was first introduced by Thistlethwaite and Campbell [59], and its theoretical framework was formally set up by Hahn, Todd, and van der Klaauw [60]. Imbens and Lemieux [61], and Lee and Lemieux [62] provide very detailed discussions and guidance on the theoretical and practical issues relating to RD. Implementation of NROAI in China can be seen as a natural experiment which meets the above pre-conditions. Below, we briefly describe this method in the context of the current study.

**Fig. 1:**
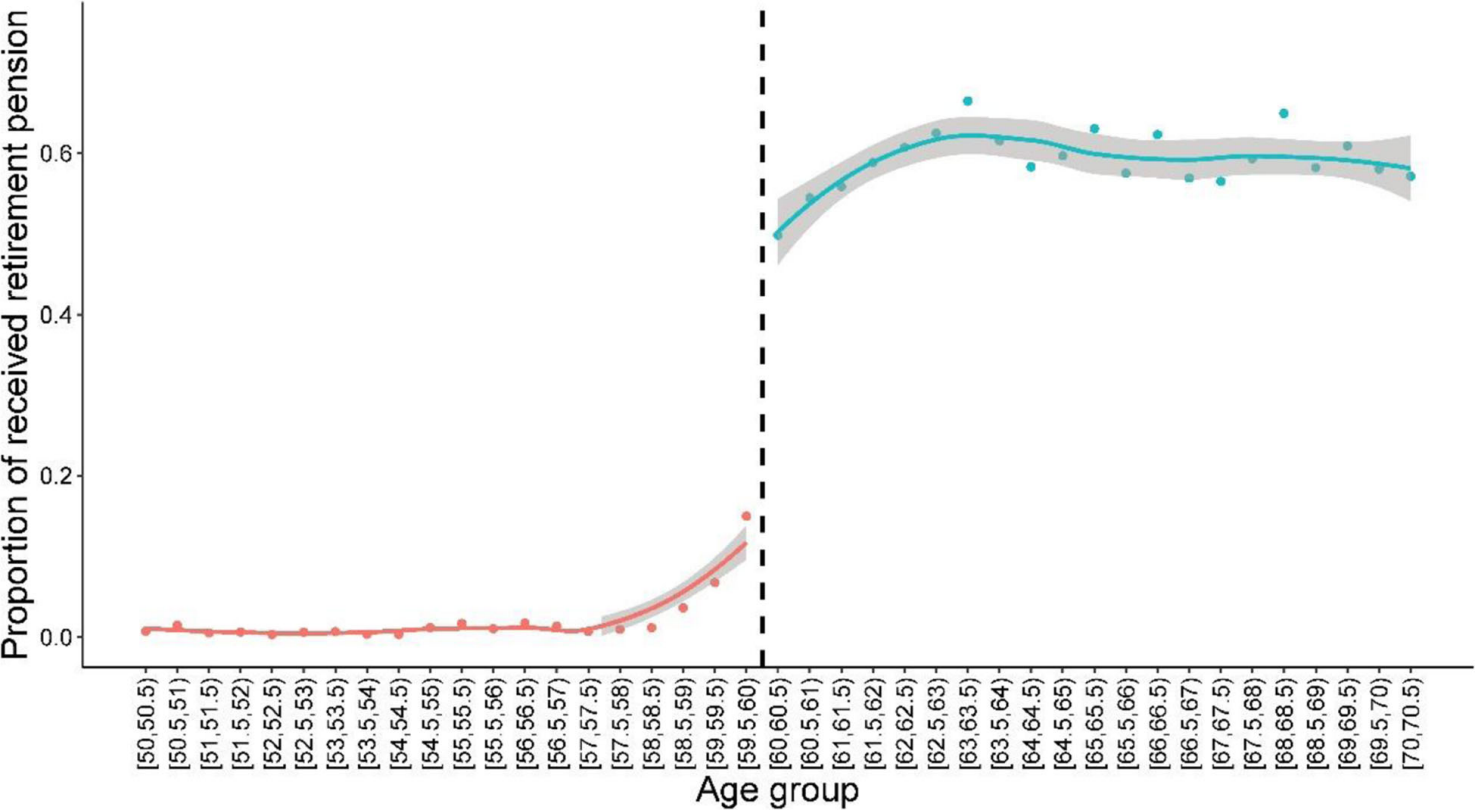
Proportion of rural elderly receiving a transferred pension

In this study, Age is defined as the force variable with a cut-off score of 60, because only residents aged 60 years or over in the rural area are covered by the NROAl. Figure 1 shows that the proportion of people who receive the pension increases sharply after 60 years. For the RD study, the data will be fitted by eq. 1.

1 $$ {Y}_i={\beta}_0+{\beta}_1\ast {pension}_i+f\left({age}_i-60\right)+\gamma \ast {X}_i+{\varepsilon}_i $$

Where *Y*_*i*_ is health service utilization;

*pension*_*i*_ is a binary variable: 1 if individual *i* receives pension, otherwise 0;

*β*_1_ is the coefficient of interest and measures the extent of transferred pension influencing the health service utilization;

*age*_*i*_ − 60 is the difference between *i* ’s actual age and the cutoff; and

*f*(*age*_*i*_ − 60) is a polynomial form of order four as suggested by Calonico [63];

*X*_*i*_ is a vector of covariates.

Figure 1 also indicates that the cut-off is not always 60, as some people obtain the pension before or after reaching this age. This is handled by adopting a variant on the RD approach known as fuzzy-RD, which uses an instrumental variable to estimate *pension*_*i*_, as shown in eq. 2.

2 $$ {pension}_i={\alpha}_0+{\alpha}_1\ast {D}_i+f\left({age}_i-60\right)+\delta \ast {X}_i+{\omega}_i $$

Where *D*_*i*_ is the instrumental variable, taking the value 1 if individual *i* is aged 60 or over, and 0 otherwise.

### Analysis two: segmented regression

To assess what level of pension is sufficient to increase recipients’ health service utilization, segmented regression was used. This approach examines the relationships between a response and one or more explanatory variables, which are piecewise linear, represented by two or more straight lines connected at unknown values [64], usually referred as breakpoints. Simply speaking, the mathematical equation of a segmented regression is shown as:

3 $$ {Y}_i={\beta}_0+{\beta}_1\ast {pension\ income}_i+{\beta}_2\ast {\left({pension\ income}_i-\uppsi \right)}_{+}+\gamma \ast {X}_i+{\varepsilon}_i $$

Where ψ is the unknown breakpoint; (*pension income*_*i*_ − ψ)_+_ = (*pension income*_*i*_ − ψ) × *I*(*pension income*_*i*_ > ψ); *I*(*pension income*_*i*_ > ψ) is equal to one when the statement is true, zero otherwise; and *X*_*i*_ refers to the covariates in eq. (1) and (2).

A detailed discussion of segmented regression, including consideration of non-linear relationships, multiple breakpoints, and hypothesis testing, has been reported elsewhere [64].

To simplify the problem in question, this study considers one or two breakpoints, and reports the results for two breakpoints if both are significant. To avoid reverse causation and omitted variable bias, we followed the suggestion of Cheng et al. [65] and Chen et al. [66], and took pension duration as an instrumental variable for pension income. This was calculated as the time between the survey year and the year the participant begin to receive the pension.

Equations (1), (2), and (3) were fitted using the general linear model. For binary outcomes, the binomial distribution was used as the link, and for count data the negative binomial distribution. All analysis was done in R (3.6.0). We took *p* < 0.05 as the level for determining statistics significance.

## Results

### Basic descriptive results

Descriptive statistics of the study sample are shown in Table 1. About 34% of the sample were illiterate, while 44% had attended or finished primary school. Those who had attended middle school or above comprised around 22%. About 16% of participants were living alone. The average ADL score (maximum value 36) was 21.04. The average self-rated health status score (maximum 5) was 2.41. About 37% of participants reported pain and about 70% suffered at least one chronic condition. The average annual household income per head was 15,856 CNY (2053 EUR or 2299 USD). About 31% received a retirement pension and the average pension amount was 71.29 CNY (9.2 EUR or 10.3 USD) per month. Around 22% of the sample utilized outpatient service, and the average number of visits was 2.35 among users. Almost 13% of participants utilized inpatient services, and the average number of episodes was 1.53 among users. When feeling ill, about 45% chose to self-treat, with around 35% purchasing over-the-counter medicine and around 10% purchasing traditional Chinese medicines.

**Table 1:** descriptive statistics for the variables in the analysis^a^

	Total (***n*** = 14,922)	Low income (***n*** = 3672)	Low-middle income (***n*** = 3727)	Middle-high income (***n*** = 3807)	High income (***n*** = 3716)
**Gender**					
Male (=yes)	44.96	42.91	43.91	47.29	45.66
**Education**					
Illiterate (=yes)	34.04	34.75	35.90	34.25	30.87
Primary school (=yes)	43.63	43.95	42.07	45.52	43.35
Middle school or above (=yes)	22.33	21.30	22.03	20.23	25.78
**Live alone** (=yes)	16.07	17.73	15.51	16.42	14.00
**Activities of Daily Living (ADL)**	21.04(6.55)	20.72(6.7)	20.97(6.55)	20.69(6.93)	21.83(5.77)
**Self-rated health status**	2.41(1.04)	2.45(1.07)	2.38(1.02)	2.33(1.04)	2.47(1.03)
**Pain** (=yes)	37.19	32.65	38.10	42.47	35.81
**Chronic** (=yes)	69.32	63.92	70.29	73.76	69.09
**Household income per capital per year**					
In CNY	15,856.19(82,270.62)	12.94(31.67)	603.6(345.96)	6096.62(3647.07)	56,808.14(157,843.57)
In EUR	2052.74(10,650.75)	1.68(4.1)	78.14(44.79)	789.27(472.15)	7354.38(20,434.43)
In USD	2299.15(11,929.24)	1.88(4.59)	87.52(50.16)	884.01(528.83)	8237.18(22,887.32)
**Pension** (=yes)	30.71	32.33	34.72	30.51	25.24
**Pension income per capital per month**					
In CNY	71.29(34.15)	75.39(38.18)	68.89(25.88)	68.46(29.51)	73.60(44.00)
In EUR	9.23(4.42)	9.76(4.94)	8.92(3.35)	8.86(3.82)	9.53(5.7)
In USD	10.34(4.95)	10.93(5.54)	9.99(3.75)	9.93(4.28)	10.67(6.38)
**Outpatient**					
Contact decision (=yes)	22.45	20.06	22.72	24.10	22.97
Frequency decision	2.35(2.64)	2.37(2.99)	2.28(1.98)	2.35(2.57)	2.32(2.57)
**Inpatient**					
Contact decision (=yes)	12.87	13.16	13.18	13.29	11.74
Frequency decision	1.53(1.31)	1.59(1.48)	1.5(1.27)	1.53(1.14)	1.5(1.3)
**Self-treatment**					
Contact decision (=yes)	45.12	48.77	45.67	42.19	43.76
Over-the-counter medicines (=yes)	35.28	32.41	35.31	37.48	36.22
Traditional Chinese medicines (=yes)	9.71	9.23	9.50	10.98	9.07
Tonic or health supplement (=yes)	5.23	5.58	4.86	4.94	5.62
Health care equipment (=yes)	0.46	0.52	0.46	0.42	0.46

^a^data is present in percentage or mean (sd). Income group is divided based on inter-quartile range and median. Income or pension was present in CNY (EUR or USD) transferred by the average exchange rate in 2019: 1 CNY = 0.12946 EUR or 0.145 USD

The distribution of the above results were highly diverse across different income groups. Low education level, living alone, low score of ADL, and low self-rated health status were mainly associated with those on low income. The gap in household disposable income per capita per year averaged 56,808 CNY (7354 EUR or 8237 USD) in the high-income group versus only 13 CNY (1.7 EUR or 1.9 USD) in the low-income group. Reports of body pain and chronic illness are highest in low-middle (38.10 and 70.29%) and middle-high income groups (42.47 and 73.76%).

### Estimation of pension effects

Table 2 presents the estimated impact of a pension on health service utilization. Overall, the pension does not influence use of outpatient services (OR = 1.044, 95% 0.956–1.140), but low-income recipients who received a pension are 1.219 times more likely to access these services. Receiving a pension results in a significant overall increase in use of inpatient services (OR = 1.237, 95% 1.108–1.381), and sub-income group analysis indicates that this is the case for low income (OR = 1.269, 95% 1.020–1.579) and middle-high income people (OR = 1.387, 95% 1.114–1.726). Pension effects on the frequency of outpatient or inpatient service use were non-significant both overall and for income groups.

**Table 2:** estimated effects of retirement pension on health service utilization^a^

	Total	Low income	Low-middle income	Middle-high income	High income
**Outpatient**					
Contact decision	1.044 [0.956, 1.140]	1.219* [1.018, 1.460]	0.974 [0.819, 1.159]	1.065 [0.893, 1.269]	0.975 [0.807, 1.176]
Frequency decision	1.034 [0.982, 1.089]	1.093 [0.983, 1.215]	0.990 [0.893, 1.098]	1.027 [0.927, 1.137]	1.005 [0.898, 1.123]
**Inpatient**					
Contact decision	1.237*** [1.108, 1.381]	1.269* [1.020, 1.579]	1.170 [0.944, 1.451]	1.387** [1.114, 1.726]	1.141 [0.891, 1.457]
Frequency decision	0.956 [0.879, 1.041]	0.940 [0.798, 1.109]	0.969 [0.819, 1.147]	0.995 [0.837, 1.183]	0.855 [0.700, 1.041]
**Self-treatment**^b^					
Contact decision	1.056 [0.981, 1.137]	1.207* [1.046, 1.393]	1.112 [0.963, 1.286]	0.872. [0.749, 1.016]	1.190* [1.014, 1.397]
Over-the-counter medicines	1.058 [0.98, 1.142]	1.208* [1.037, 1.407]	1.099 [0.945, 1.278]	0.847* [0.725, 0.991]	1.206* [1.024, 1.419]
Traditional Chinese medicines	1.242** [1.083, 1.423]	1.452* [1.094, 1.932]	1.201 [0.917, 1.573]	1.068 [0.814, 1.396]	1.456* [1.079, 1.955]
Tonic or health supplement	1.247* [1.039, 1.495]	1.101 [0.772, 1.572]	1.354 [0.938, 1.955]	1.327 [0.899, 1.945]	1.292 [0.884, 1.869]

^a^Data is present in effect value (sd), for contract decision the data is present in *e*^*effect value*^ (sd)

^b^We don’t estimated the results for “Health care equipment”, as the number of people who utilized it is too small

*** *p* < 0.001, ** *p* < 0.01, * *p* < 0.05,. *p* < 0.1

Receiving a retirement pension had no significant effect on self-treatment overall, but significantly increased the likelihood of utilizing Chinese traditional medicines (OR = 1.242, 95% 1.083–1.423), and tonics or health supplements (OR = 1.247, 95% 1.039–1.495). Subgroup analysis indicated that a pension increased the likelihood of utilizing self-treatment among low-income people (OR = 1.207, 95% 1.046–1.393), both for over-the-counter medicines (OR = 1.208, 95% 1.037–1.407) and traditional Chinese medicines (OR = 1.452, 95% 1.094–1.932). Similar effects were found for the high-income group. On the contrary, a pension reduced the likelihood of using over-the-counter medicines among middle-income people (OR = 0.847, 95% 0.725–0.991).

#### Robustness test

In the above results the bandwidth was set to ten. Only individuals aged between 50 and 70 were included. To examine the robustness of these findings, a sensitivity analysis with different age bandwidths was performed. The results are shown in Table 3, which indicates that the use of different age bandwidths had no significant impact on the main outcomes.

**Table 3:** Robust test of retirement pension on health service utilization by different bandwidth^a^

	Bandwidth^b^	Total	Low income	Low-middle income	Middle-high income	High income
**Outpatient**						
Contact decision	bw = 3	1.081 [0.926, 1.26]	1.502** [1.108, 2.035]	1.24 [0.928, 1.651]	0.9 [0.625, 1.277]	0.743 [0.512, 1.057]
	bw = 5	1.038 [0.921, 1.168]	1.308* [1.027, 1.667]	1.018 [0.81, 1.275]	1.041 [0.809, 1.335]	0.898 [0.692, 1.159]
	bw = 7	0.998 [0.904, 1.101]	1.271* [1.04, 1.555]	0.93 [0.767, 1.127]	0.969 [0.791, 1.184]	0.899 [0.726, 1.109]
	bw = IK	1.003 [0.914, 1.101]	1.237* [1.028, 1.489]	0.922 [0.762, 1.114]	1.054 [0.82, 1.349]	0.895 [0.723, 1.105]
Frequency decision	bw = 3	0.949 [0.864, 1.039]	0.968 [0.806, 1.161]	0.892 [0.75, 1.059]	0.983 [0.785, 1.22]	0.968 [0.773, 1.199]
	bw = 5	1.026 [0.957, 1.099]	1.103 [0.953, 1.277]	0.911 [0.797, 1.041]	1.116 [0.965, 1.289]	0.955 [0.816, 1.113]
	bw = 7	1.209 [0.847, 1.701]	1.031 [0.53, 1.957]	0.962 [0.858, 1.078]	1.095 [0.975, 1.228]	1.051 [0.925, 1.192]
	bw = IK	1.041 [0.988, 1.097]	1.084 [0.969, 1.212]	0.982 [0.881, 1.095]	0.976 [0.889, 1.073]	1.079 [0.955, 1.217]
**Inpatient**						
Contact decision	bw = 3	1.191. [0.98, 1.443]	1.526* [1.047, 2.222]	1.151 [0.788, 1.665]	1.352* [1.068, 1.713]	1.052 [0.666, 1.61]
	bw = 5	1.203* [1.038, 1.393]	1.301. [0.969, 1.748]	1.163 [0.881, 1.531]	1.275* [1.002, 1.621]	1.081 [0.772, 1.497]
	bw = 7	1.183** [1.046, 1.338]	1.297* [1.014, 1.66]	1.073 [0.848, 1.358]	1.355* [1.037, 1.771]	1.101 [0.833, 1.446]
	bw = IK	1.212*** [1.091, 1.347]	1.293* [1.035, 1.618]	1.073 [0.846, 1.359]	1.336* [1.063, 1.679]	1.121 [0.865, 1.449]
Frequency decision	bw = 3	0.88. [0.758, 1.019]	0.816 [0.62, 1.071]	0.916 [0.674, 1.235]	0.839 [0.577, 1.187]	0.825 [0.575, 1.154]
	bw = 5	0.919 [0.821, 1.028]	0.926 [0.743, 1.156]	0.864 [0.696, 1.071]	0.956 [0.75, 1.215]	0.877 [0.668, 1.142]
	bw = 7	0.918. [0.835, 1.009]	0.926 [0.771, 1.112]	0.935 [0.779, 1.122]	0.955 [0.781, 1.165]	0.847. [0.709, 1.011]
	bw = IK	0.925 [0.841, 1.016]	0.872. [0.749, 1.017]	0.969 [0.819, 1.147]	0.981 [0.819, 1.176]	0.811. [0.651, 1.006]
**Self-treatment**						
Contact decision	bw = 3	1.108 [0.975, 1.261]	1.666** [1.171, 2.347]	1.269. [0.992, 1.625]	0.921 [0.69, 1.232]	1.977* [1.032, 3.771]
	bw = 5	1.078 [0.977, 1.189]	1.273* [1.05, 1.542]	1.097 [0.91, 1.323]	0.854 [0.69, 1.056]	1.302* [1.051, 1.616]
	bw = 7	1.074. [0.99, 1.165]	1.251** [1.067, 1.467]	1.14 [0.973, 1.336]	0.876 [0.738, 1.041]	1.207* [1.012, 1.441]
	bw = IK	1.084. [0.996, 1.181]	1.231* [1.038, 1.461]	1.136 [0.95, 1.359]	0.887 [0.757, 1.039]	1.27* [1.057, 1.527]
Over-the-counter medicines	bw = 3	1.054 [0.921, 1.205]	1.181 [0.906, 1.538]	1.232 [0.954, 1.59]	0.76* [0.584, 0.98]	1.265** [1.065, 1.502]
	bw = 5	1.061 [0.958, 1.175]	1.255* [1.021, 1.542]	1.122 [0.924, 1.362]	0.821. [0.658, 1.021]	1.27* [1.043, 1.545]
	bw = 7	1.044 [0.682, 1.582]	1.251** [1.056, 1.483]	1.137 [0.965, 1.34]	0.79* [0.627, 0.99]	1.289** [1.078, 1.541]
	bw = IK	1.076. [0.993, 1.167]	1.228** [1.052, 1.434]	1.131 [0.962, 1.33]	0.785* [0.645, 0.953]	1.293** [1.081, 1.545]
Traditional Chinese medicines	bw = 3	1.341** [1.091, 1.642]	1.092* [1.004, 1.188]	1.124 [0.954, 1.325]	1.349 [0.876, 2.032]	1.691* [1.07, 2.607]
	bw = 5	1.275** [1.082, 1.499]	1.468* [1.078, 2.004]	1.195 [0.871, 1.635]	1.286 [0.925, 1.775]	1.535* [1.065, 2.188]
	bw = 7	1.182** [1.045, 1.336]	1.385* [1.06, 1.815]	1.137 [0.89, 1.455]	1.072 [0.844, 1.359]	1.425** [1.087, 1.863]
	bw = IK	1.279** [1.093, 1.494]	1.388* [1.062, 1.817]	1.233 [0.92, 1.652]	1.292 [0.927, 1.788]	1.451* [1.077, 1.943]
Tonic or health supplement	bw = 3	1.389* [1.045, 1.833]	1.423 [0.814, 2.481]	1.421 [0.83, 2.395]	1.214 [0.619, 2.228]	1.326 [0.676, 2.431]
	bw = 5	1.258* [1.009, 1.563]	1.26 [0.81, 1.961]	1.313 [0.858, 1.999]	1.195 [0.743, 1.89]	1.217 [0.755, 1.921]
	bw = 7	1.551* [1.042, 2.295]	1.099 [0.8, 1.511]	1.131 [0.808, 1.586]	1.219 [0.866, 1.71]	1.109 [0.789, 1.552]
	bw = IK	1.205* [1.012, 1.432]	1.098 [0.795, 1.519]	1.217 [0.812, 1.815]	1.183 [0.842, 1.656]	1.233 [0.856, 1.762]

^a^(data is present in effect value (sd), for contract decision the data is present in *e*^*effect value*^ (sd)

^b^bw = IK means the bandwidth is the optimal bandwidth calculated by Imbens-Kalyanaraman method [67]

*** *p* < 0.001, ** *p* < 0.01, * *p* < 0.05,. *p* < 0.1

#### McCracy test for manipulation of the force variable (age)

In practice, people may falsely report their age to gain a pension. This will undermine the assumed continuity of the conditional expectation of counterfactual outcomes in the force variable, and adversely affects the validity of the above results. The McCracy test, which tests the continuity of the force variable’s density function [68], was conducted to check the possibility of age manipulation. The results of the McCracy test show that with 95% power this study can accept the hypothesis that the density of the age variable is continuous around the cut-off of 60 for all samples and income groups. Detailed results are provided in the supplementary materials, Additional file 1: appendix 2-1 to 2-3.

#### Testing for balanced covariates

To build the causal relationship, RD relies on the condition that receiving a pension is the only factor which has a step change before and after the age of 60. In other words, it means that other covariates have to remain stable or balanced. One way to do this is to use eq. (1) and (2) but set the dependent variable as the covariate we want to test [61, 62]. A non-significant result will support the hypothesis that the covariate is balanced. The results are provided in the supplementary materials, Additional file 1: appendix 3-1 to 3-3, and indicate that all the coefficient are non-significant at the 5% level.

### Segmented effects of transferred pension income

**Fig. 2:**
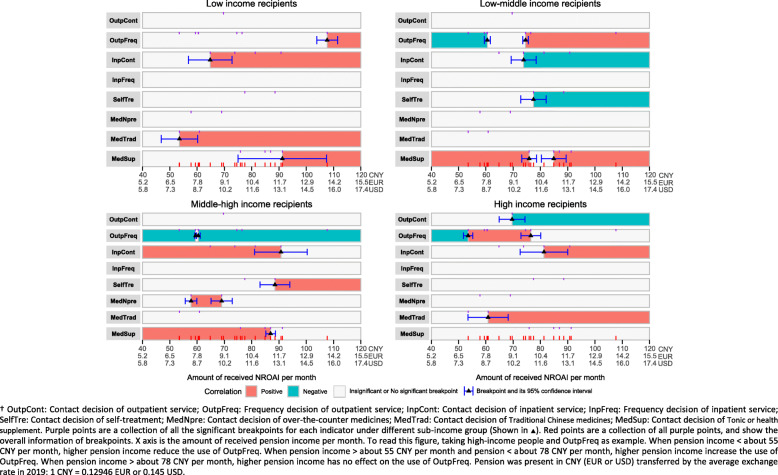
Segmented regression of transferred cash on health service utilization

Figure 2 shows that there are several breakpoints for a pension that promotes increased health service utilization. Overall, most are located in the range 55–95 CNY (7.1–12.3 EUR or 8.0–13.8 USD) per month. For low-income people, there is a low breakpoint, around 55–65 CNY (7.1–8.4 EUR or 8.0–9.4 USD) per month, for utilization of inpatient services and Chinese traditional medicine, but a high breakpoint, around 90–110 CNY (11.7–14.2 EUR or 13.1–16.0 USD) per month, for outpatient services and tonics or health supplements. For low-middle income people, too low a pension, under some 60 CNY (7.8 EUR or 8.7 USD) per month, promotes reduced use of outpatient services, while a pension up to about 75 CNY (9.7 EUR or 10.9 USD) per month encourages increased use. A higher pension discourages the use of inpatient services and self-treatment for this group, while both low and high pension levels increase consumption of tonics and health supplements. For the middle-high income group, a lower level of pension, under some 90 CNY (11.7 EUR or 13.1 USD) per month, results in increased use of inpatient services and tonics and health supplements, while a higher level, above 90 CNY (11.7 EUR or 13.1 USD), promotes their use of self-treatment. For high-income people, a lower breakpoint, around 60 CNY (7.8 EUR or 8.7 USD) per month, encourages increased use of Chinese traditional medicine, and a higher breakpoint, around 82 CNY (10.6 EUR or 11.9 USD) per month, greater use of inpatient services. Additionally, a higher pension, above some 70 CNY (9.1 EUR or 10.2 USD) per month, appears to reduce the overall use of outpatient services.

## Discussion

As far as we are aware, this is the first study in China combining the two topics of pensions and health service utilization using a rigorous impact evaluation methodology. We focused on not only outpatient and inpatient services, the common approach to measure health service utilization, but also self-treatment, a high prevalence but less focused healthcare seeking behaviour [38]. In addition, we not only estimated the overall impact of a pension on our population sample, but also on distinct income groups. More importantly, we further explored what level of pension would be enough to influence health service utilization.

Our findings partly support our hypothesis that a pension will facilitate people using health services, especially the poor. By comparing the disposable income and the pension income shown in Table 1, we inferred that poor people would be more sensitive to additional cash transfers by retirement pension, which would improve the affordability of outpatient services, non-prescription medicines, and traditional medicines. Results in Table 2 supported this inference to certain extent, as there was no significant effect of retirement pension on the frequency of outpatient visits and the utilization of tonics or health supplements. Multiple outpatient visits would imply substantially greater expenditures, and consumption of tonics and supplements may be seen by the poor as non-essential spending.

Our findings indicating that a pension promotes increased use of inpatient services by low-income people is in line with previous evidence, which indicates that the poor are more sensitive to the costs of transportation, subsistence and co-payments [22], which often impede their access to health services [17, 21, 22]. In China, these costs of access to inpatient services are typically considerably higher than those for outpatient services. Our finding indicate that a pension can indeed offset these barriers.

One interesting finding of our study is that the healthcare seeking behaviour of high-income people is also influenced by a pension but only in term of increased use of self-treatment, including over-the-counter medicines and traditional Chinese medicines. Why the pension increases self-treatment but does not promote their increased use of outpatient or inpatient services, or increased purchasing of tonics or health supplements is not clear, given that the pension may be seen as a marginal additional to their existing high level of income. Our focus on a rural population would seem to exclude explanations relating to changes in their social environment, availability of free time, or reduction of regular wages or salaries. Future analysis on this topic through a qualitative approach will be needed to address this knowledge gap.

In this study, we found that a pension had no significant effects on the frequency of inpatient or outpatient service use. There are three possible explanations. First, the value of the pension may be too low to support frequent service utilization; second, having decided to utilize a service, having the pension may encourage an individual to spend more on each visit. Third, recipients may choose to use the pension income to seek higher quality services. However, we were unable to determine the relative importance of these options.

One significant practical contribution of our study is the exploration into the level of pension required to promote increased use of health services. The breakpoints for the pension to play this role were mostly located in the range 55–95 CNY (7.1–12.3 EUR or 8.0–13.8 USD) per month, though about 110 CNY (14.2 EUR or 16.0 USD) per month was needed to encourage increased use of outpatient services by low-income people. The breakpoints in Fig. 2 not only show the sensitivity of different income groups to the value of a pension and how they substitute services in response to different pension levels, but more importantly imply that recipients’ response to a given level of pension may be influenced by their health literacy. This implication is seen especially among low-middle and middle-high income groups. When the pension is low, they will reduce the utilization of formal outpatient services, preferring to purchase tonics or health supplements. That was clearly not an intended outcome of the pension policy intervention, and is a reminder to policymakers that the response to a given intervention may often be more complex than expected.

The results in Fig. 2 also reveal a limitation of our study; that existing pension values lack enough variation to explore the breakpoint for decisions relating to the frequency of health service utilization. Including data from other counties, both richer and poorer, would have been informative. Two other limitations can be noted. First, although the focus on rural residents allowed us to control possible biases from the change of social circumstances, availability of free time, and reduction of regular wages or salaries, it limits the relevance of our findings for urban populations; second, we did not explore the effects of the interaction between pensions and health insurance on health service utilization. This is an essential policy concern, and needs further study.

## Conclusions

In summary, using a nationally representative sample survey, we adopted a quasi-experimental research design and estimated the effects of a pension on older people’s health service utilization. In addition, we also did segmented regression and explored what level of pension would be enough to influence recipients’ health service utilization. This study found that: first, a pension facilitates low-income people to utilize outpatient services; second, it promotes the use of inpatient services by low-income and middle-high-income people; third, a pension has no effect on the number of outpatient visits or inpatient admissions for those utilizing these services; fourth, it encourages both low-income people and high-income people to make greater use of self-treatment, specifically non-prescription medicines and traditional medicines; fifth, the levels of pension required to promote recipients’ health service utilization for different income groups lie mainly in the range of 55–95 CNY (7.1–12.3 EUR or 8.0–13.8 USD) per month. Our finding imply that a pension can indeed offset the cost barriers associated with transportation, subsistence and copayments. The information our study presents can allow economists and decision makers to model pension policies and their potential role in meeting health care needs with greater precision.

## Supplementary information

**Additional file 1: Appendix 1.** the definition or measurement of the Covariate variables. **Appendix 2**-**1.** McCracy test for manipulation of age (all samples) by different bandwidth. Appendix 2-2 McCracy test for manipulation of age (outpatient = yes) by different bandwidth. **Appendix 2**-**3.** McCracy test for manipulation of age (inpatient = yes) by different bandwidth. **Appendix 3**-**1** balance test for covariates (all samples) by different bandwidth and cutoff. **Appendix 3**-**2.** balance test for covariates (outpatient = yes) by different bandwidth and cutoff. **Appendix 3**-**3.** balance test for covariates (inpatient = yes) by different bandwidth and cutoff.

## Data Availability

The data is publicly accessible from The China Health and Retirement Longitudinal Study (CHARLS).

## Abbreviations

ADL: Activities of Daily Living
CCTs: Conditional Cash Transfers
CHARLS: China Health and Retirement Longitudinal Study
CNY: China Yuan
LMIC: Low- and Middle-Income Countries
NCMS: New Rural Cooperative Medical System
NROAI: New Rural Old Age Insurance
OR: Odd Ratio
RD: Regression Discontinuity
UCTs: Unconditional Cash Transfers
USD: USA dollar
WHO: World Health Organization
