# Supplementary material for: How and to what extent can pensions facilitate increased use of health services by older people: evidence from social pension expansion in rural China
Source: BMC Health Serv Res. 2020 Nov 4;20:1008. doi: 10.1186/s12913-020-05831-0 (PMC7640495; doi:10.1186/s12913-020-05831-0)
Supplement: Supplementary file 1 — Additional file 1: Appendix 1. the definition or measurement of the Covariate variables. Appendix 2-1. McCracy test for manipulation of age (all samples) by different bandwidth. Appendix 2-2 McCracy test for manipulation of age (outpatient = yes) by different bandwidth. Appendix 2-3. McCracy test for manipulation of age (inpatient = yes) by different bandwidth. Appendix 3-1 balance test for covariates (all samples) by different bandwidth and cutoff. Appendix 3-2. balance test for covariates (outpatient = yes) by different bandwidth and cutoff. Appendix 3-3. balance test for covariates (inpatient = yes) by different bandwidth and cutoff. [file 12913_2020_5831_MOESM1_ESM.docx]

#

# Appendix 1 the definition or measurement of the Covariate variables

- **Household income per capital per year (CNY)**

Per capita disposable income was constructed from the yearly total disposable household income divided by the number of household members. Household income includes including the aggregate of household income from production, wage incomes of household members, transfer income (remittances, welfare) and property income (interest, rent).

- **Live alone**

Following conditions will treated as live alone.

- Married but not living with spouse temporarily for reasons such as work
- Separated
- Divorced
- Widowed
- Never married
- **Activities of Daily Living (ADL)**

ADL is measured using following nine questions. For each questions, one stands for “I can not do it”, and four stands for “No, I don’t have any difficulty”.

- Do you have any difficulty with running or jogging about 1 Km?
- Do you have difficulty walking 1 km?
- Do you have difficulty walking 100 metres?
- Do you have difficulty getting up from a chair after sitting for a long period?
- Do you have difficulty climbing several flights of stairs without resting?
- Do you have difficulty stooping, kneeling, or crouching?
- Do you have difficulty reaching or extending your arms above shoulder level? (He/she is regarded as not having difficulty only if he/she can extend both of his/her arms, otherwise he/she is regarded as having difficulty.)
- Do you have difficulty lifting or carrying weights over 10 jin, lIKe a heavy bag of groceries?
- Do you have difficulty picking up a small coin from a table?
- **Pain**

Whether feeling pain is got though the question: On what part of your body do you feel pain?

- Head (Headache)
- Shoulder
- Arm
- Wrist
- Fingers
- Chest
- Stomach (Stomachache)
- Back
- Waist
- Buttocks
- Leg
- Knees
- Ankle
- Toes
- Neck
- **Chronic**

Whether has chronic is got though the question: Have you been diagnosed with conditions listed below by a doctor?

- Hypertension
- Dyslipidemia (elevation of low density lipoprotein, triglycerides (TGs),and total cholesterol, or a low high density lipoprotein level)
- Diabetes or high blood sugar
- Cancer or malignant tumor (excluding minor skin cancers)
- Chronic lung diseases, such as chronic bronchitis , emphysema ( excluding tumors, or cancer)
- Liver disease (except fatty liver, tumors, and cancer)
- Heart attack, coronary heart disease, angina, congestive heart failure, or other heart problems
- Stroke
- Kidney disease (except for tumor or cancer)
- Stomach or other digestive disease (except for tumor or cancer)
- Emotional, nervous, or psychiatric problems
- Memory-related disease
- Arthritis or rheumatism
- Asthma

**Self-rated health status**

This got through question: How would you rate your health status? With one stand for poor and five stand for excellent.

# Appendix 2-1 McCracy test for manipulation of age (all samples) by different bandwidth

| **Income group** | **Item** | **bw = no constrain** | **bw = 3** | **bw = 5** | **bw = 7** | **bw = IK** |
| --- | --- | --- | --- | --- | --- | --- |
| Total | Discontinuity | -0.02 | 0.005 | -0.028 | -0.049 | -0.042 |
|  | z-value | -0.235 | 0.05 | -0.369 | -0.772 | -0.61 |
|  | p-value | 0.814 | 0.96 | 0.712 | 0.44 | 0.542 |
| Low | Discontinuity | 0.012 | 0.012 | 0.009 | 0.041 | 0.022 |
|  | z-value | 0.06 | 0.055 | 0.059 | 0.323 | 0.155 |
|  | p-value | 0.952 | 0.956 | 0.953 | 0.747 | 0.877 |
| Low-middle | Discontinuity | 0.09 | 0.218 | 0.079 | 0.027 | 0.046 |
|  | z-value | 0.577 | 1.054 | 0.518 | 0.212 | 0.331 |
|  | p-value | 0.564 | 0.292 | 0.604 | 0.832 | 0.741 |
|  | Discontinuity | -0.064 | -0.088 | -0.057 | -0.092 | -0.069 |
| Middle-high | z-value | -0.408 | -0.471 | -0.389 | -0.764 | -0.518 |
|  | p-value | 0.683 | 0.638 | 0.697 | 0.445 | 0.605 |
|  | Discontinuity | -0.162 | -0.186 | -0.164 | -0.183 | -0.182 |
| High | z-value | -0.984 | -0.948 | -1.055 | -1.382 | -1.277 |
|  | p-value | 0.325 | 0.343 | 0.291 | 0.167 | 0.202 |

† bw = IK means the bandwidth is the optimal bandwidth calculated by Imbens-Kalyanaraman method.

# Appendix 2-2 McCracy test for manipulation of age (outpatient = yes) by different bandwidth

| **Income group** | **Item** | **bw = no constrain** | **bw = 3** | **bw = 5** | **bw = 7** | **bw = IK** |
| --- | --- | --- | --- | --- | --- | --- |
|  | Discontinuity | -0.068 | -0.024 | -0.061 | -0.066 | -0.077 |
| Total | z-value | -0.452 | -0.118 | -0.388 | -0.497 | -0.807 |
|  | p-value | 0.651 | 0.906 | 0.698 | 0.619 | 0.42 |
|  | Discontinuity | 0.055 | -0.04 | -0.074 | -0.406 | -0.078 |
| Low | z-value | 0.614 | -0.227 | -0.518 | -1.505 | -0.402 |
|  | p-value | 0.537 | 0.82 | 0.604 | 0.132 | 0.687 |
|  | Discontinuity | 0.315 | 0.303 | 0.215 | 0.138 | 0.158 |
| Low-middle | z-value | 0.803 | 0.709 | 0.664 | 0.513 | 0.851 |
|  | p-value | 0.422 | 0.479 | 0.507 | 0.608 | 0.395 |
|  | Discontinuity | 0.319 | 0.363 | 0.279 | 0.069 | -0.245 |
| Middle-high | z-value | 0.939 | 0.863 | 0.852 | 0.258 | -1.324 |
|  | p-value | 0.348 | 0.388 | 0.394 | 0.796 | 0.185 |
|  | Discontinuity | -0.03 | -0.008 | -0.053 | -0.035 | -0.131 |
| High | z-value | -0.087 | -0.022 | -0.177 | -0.129 | -0.644 |
|  | p-value | 0.931 | 0.983 | 0.859 | 0.897 | 0.52 |

† bw = IK means the bandwidth is the optimal bandwidth calculated by Imbens-Kalyanaraman method

# Appendix 2-3 McCracy test for manipulation of age (inpatient = yes) by different bandwidth

| **Income group** | **Item** | **bw = no constrain** | **bw = 3** | **bw = 5** | **bw = 7** | **bw = IK** |
| --- | --- | --- | --- | --- | --- | --- |
|  | Discontinuity | -0.017 | -0.05 | -0.112 | -0.01 | 0.054 |
| Total | z-value | -0.146 | -0.205 | -0.406 | -0.044 | 0.289 |
|  | p-value | 0.885 | 0.837 | 0.685 | 0.965 | 0.772 |
|  | Discontinuity | 0.022 | 0.534 | 0.543 | 0.388 | 0.41 |
| Low | z-value | 0.155 | 0.98 | 0.971 | 0.862 | 1.075 |
|  | p-value | 0.876 | 0.327 | 0.331 | 0.389 | 0.282 |
|  | Discontinuity | 0.446 | 0.555 | 0.294 | 0.286 | 0.29 |
| Low-middle | z-value | 0.776 | 0.832 | 0.667 | 0.78 | 0.89 |
|  | p-value | 0.438 | 0.406 | 0.505 | 0.435 | 0.374 |
|  | Discontinuity | -0.149 | -0.205 | -0.093 | -0.07 | -0.098 |
| Middle-high | z-value | -0.25 | -0.307 | -0.19 | -0.176 | -0.286 |
|  | p-value | 0.803 | 0.759 | 0.85 | 0.861 | 0.775 |
|  | Discontinuity | 0.353 | 0.336 | 0.339 | 0.45 | 0.268 |
| High | z-value | 0.533 | 0.454 | 0.603 | 0.893 | 0.596 |
|  | p-value | 0.594 | 0.65 | 0.546 | 0.372 | 0.551 |

† bw = IK means the bandwidth is the optimal bandwidth calculated by Imbens-Kalyanaraman method.

# Appendix 3-1 balance test for covariates (all samples) by different bandwidth and cutoff

| **Income group** | **Covariates** | **bw = no constrain** | **bw = 3** | **bw = 5** | **bw = 7** | **bw = IK** |
| --- | --- | --- | --- | --- | --- | --- |
| Total | Gender | 0.239(0.257) | 0.547(0.435) | 0.291(0.348) | 0.262(0.301) | 0.225(0.26) |
| Total | Education | -0.199(0.255) | 0.157(0.427) | -0.213(0.344) | -0.031(0.297) | -0.204(0.337) |
| Total | Live alone | -0.134(0.331) | -0.199(0.533) | -0.046(0.448) | -0.25(0.388) | -0.206(0.382) |
| Total | Activities of Daily Living (ADL) | 1.308(0.593) | 1.127(1.008) | 1.404(0.813) | 1.532(0.692) | 1.512(0.694) |
| Total | Pain | 0.17(0.257) | 0.236(0.436) | 0.128(0.347) | 0.103(0.3) | 0.17(0.257) |
| Total | Chronic | -0.056(0.279) | 0.042(0.453) | -0.176(0.374) | -0.164(0.328) | -0.213(0.313) |
| Total | Per household income | -1754.375(1295.885) | -516.766(1575.506) | -1630.471(1155.765) | -1731.937(1092.533) | -1754.375(1295.885) |
| Low | Gender | 0.811(0.557) | 1.773(0.942). | 0.871(0.761) | 0.437(0.652) | 0.727(0.572) |
| Low | Education | -0.064(0.53) | 0.092(0.868) | -0.46(0.722) | -0.152(0.619) | -0.343(0.681) |
| Low | Live alone | -0.519(0.613) | 0.698(1.055) | -0.195(0.831) | -0.565(0.717) | -0.519(0.613) |
| Low | Activities of Daily Living (ADL) | 1.478(1.324) | 2.147(2.143) | 1.839(1.794) | 1.508(1.512) | 1.478(1.324) |
| Low | Pain | 0.511(0.539) | -0.515(0.882) | -0.583(0.715) | -0.154(0.621) | 0.511(0.539) |
| Low | Chronic | 0.206(0.577) | -0.148(0.924) | 0.184(0.772) | 0.181(0.691) | 0.138(0.629) |
| Low | Per household income | 128.422(64.049) | 182.203(105.961) | 191.744(86.041) | 160.849(75.686) | 128.422(64.049) |
| Low-middle | Gender | -0.073(0.55) | 0.857(1.126) | 0.951(0.802) | 0.206(0.656) | 0.133(0.647) |
| Low-middle | Education | -0.341(0.536) | 1.283(1.038) | -0.007(0.769) | 0.072(0.639) | 0.157(0.701) |
| Low-middle | Live alone | 0.418(0.728) | -0.884(1.456) | 0.267(1.169) | 0.366(0.904) | 0.11(0.747) |
| Low-middle | Activities of Daily Living (ADL) | 0.652(1.285) | -1.315(2.451) | -1.206(1.81) | 0.335(1.524) | 0.407(1.371) |
| Low-middle | Pain | -0.444(0.527) | 0.293(0.984) | -0.41(0.74) | 0.227(0.623) | -0.444(0.527) |
| Low-middle | Chronic | -0.977(0.616) | -2.066(1.373) | -2.026(0.957)* | -1.162(0.746) | -1.263(0.795) |
| Low-middle | Per household income | 252.482(267.796) | 158.582(528.843) | 436.676(390.817) | 375.677(318.887) | 479.183(335.595) |
| Middle-high | Gender | 0.314(0.489) | -0.315(0.778) | -0.499(0.642) | 0.207(0.562) | 0.404(0.555) |
| Middle-high | Education | -0.422(0.487) | -1.069(0.793) | -0.862(0.644) | -0.465(0.56) | -0.422(0.487) |
| Middle-high | Live alone | 1.193(0.702). | 0.962(1.048) | 1.394(0.931) | 1.17(0.805) | 1.174(0.862) |
| Middle-high | Activities of Daily Living (ADL) | 0.571(1.072) | -0.496(1.68) | 0.891(1.438) | 1.112(1.252) | 0.757(1.149) |
| Middle-high | Pain | 0.221(0.49) | 0.645(0.803) | 1.002(0.652) | 0.369(0.565) | 0.344(0.549) |
| Middle-high | Chronic | -0.108(0.525) | -0.255(0.82) | 0.128(0.697) | 0.029(0.621) | -0.108(0.525) |
| Middle-high | Per household income | -869.264(521.048) | -613.141(847.723) | -624.481(681.947) | -840.787(592.962) | -869.264(521.048) |
| High | Gender | -0.032(0.513) | 0.194(0.886) | 0.176(0.698) | 0.231(0.598) | -0.008(0.554) |
| High | Education | -0.009(0.53) | 0.432(0.91) | 0.481(0.723) | 0.378(0.618) | 0.72(0.686) |
| High | Live alone | -1.724(0.82)* | -1.243(1.145) | -0.937(0.997) | -1.946(0.976)* | -1.724(0.82)* |
| High | Activities of Daily Living (ADL) | 1.889(1.102) | 3.146(2.078) | 2.686(1.579) | 2.375(1.317) | 1.889(1.102) |
| High | Pain | 0.142(0.553) | 0.02(0.986) | -0.094(0.777) | -0.41(0.665) | 0.031(0.584) |
| High | Chronic | 0.573(0.579) | 1.788(0.984). | 0.524(0.76) | 0.198(0.657) | 0.573(0.579) |
| High | Per household income | -3607.597(4702.105) | 3343.544(5519.788) | -1370.25(3820.76) | -3551.253(3581.87) | -3607.597(4702.105) |

† Data is present in effect value (sd), *** p < 0.001, ** p < 0.01, * p < 0.05, . p < 0.1.

‡ bw = IK means the bandwidth is the optimal bandwidth calculated by Imbens-Kalyanaraman method.

# Appendix 3-2 balance test for covariates (outpatient = yes) by different bandwidth and cutoff

| **Income group** | **Covariates** | **bw = no constrain** | **bw = 3** | **bw = 5** | **bw = 7** | **bw = IK** |
| --- | --- | --- | --- | --- | --- | --- |
| Total | Gender | 0.908(0.542). | 0.723(0.921) | 0.243(0.731) | 0.416(0.626) | 0.44(0.606) |
| Total | Education | 0.392(0.524) | 1.241(0.893) | 0.751(0.713) | 0.741(0.614) | 0.392(0.524) |
| Total | Live alone | -0.667(0.63) | -0.462(1.015) | -0.082(0.832) | -0.34(0.722) | -0.684(0.647) |
| Total | Activities of Daily Living (ADL) | 4.148(1.247) | 7.204(2.137) | 6.942(1.687) | 5.424(1.446) | 6.464(1.584) |
| Total | Pain | 0.84(0.525) | 1.887(0.917)* | 1.431(0.737). | 1.368(1.633) | 1.111(0.611). |
| Total | Chronic | -0.271(0.722) | -0.164(1.154) | 0.013(0.926) | -0.578(0.86) | -0.474(0.863) |
| Total | Per household income | -8.398(1939.041) | 1940.511(3194.013) | 642.72(2198.73) | -661.386(2346.238) | -1281.183(2203.751) |
| Low | Gender | 0.715(1.176) | -1.534(2.942) | -0.734(1.916) | -0.48(1.489) | 0.371(1.22) |
| Low | Education | -0.177(1.144) | 0.608(2.589) | 0.648(1.796) | 0.028(1.419) | -0.126(1.315) |
| Low | Live alone | -1.39(1.257) | -0.604(3.484) | 0.02(2.081) | -1.133(1.719) | -0.838(1.488) |
| Low | Activities of Daily Living (ADL) | 4.391(2.846) | 11.367(6.55) | 10.717(4.503) | 5.719(3.489) | 4.391(2.846) |
| Low | Pain | 0.048(1.155) | 0.343(2.68) | 0.817(1.98) | 0.486(1.519) | -0.172(1.312) |
| Low | Chronic | 0.937(1.834) | 53.271(32.222). | 5.031(4.029) | 2.902(2.632) | 0.937(1.834) |
| Low | Per household income | 208.503(143.7) | -186.327(350.986) | 137.68(235.632) | 142.748(188.232) | 208.503(143.7) |
| Low-middle | Gender | 0.694(1.221) | -0.7(2.361) | -0.98(1.725) | -0.6(1.409) | 0.534(1.239) |
| Low-middle | Education | 1.087(1.074) | 1.33(1.759) | 0.061(1.4) | 0.679(1.222) | 0.867(1.17) |
| Low-middle | Live alone | -0.155(1.459) | -3.656(3.168) | -0.907(2.089) | -0.779(1.693) | -1.3(1.9) |
| Low-middle | Activities of Daily Living (ADL) | 4.943(2.632) | 9.324(4.784) | 8.364(3.536) | 7.235(3.067) | 6.794(3.15) |
| Low-middle | Pain | 1.934(1.124). | 10.38(5.325). | 4.583(5.01) | 5.126(6.725) | 2.26(1.159). |
| Low-middle | Chronic | -3.789(3.073) | -1624.062(1058.109) | -7.796(10.727) | -6.636(5.887) | -10.475(9.378) |
| Low-middle | Per household income | 313.805(547.993) | 622.224(925.992) | 1070.901(728.395) | 665.992(607.091) | 686.148(621.661) |
| Middle-high | Gender | 2.08(1.184). | 1.698(2.5) | 0.161(1.631) | 1.191(1.371) | 1.06(1.499) |
| Middle-high | Education | -0.161(1.088) | 0.184(1.989) | 0.071(1.539) | -0.315(1.271) | 0.435(1.328) |
| Middle-high | Live alone | 2.279(1.449) | 1.146(2.198) | 2.186(2.119) | 2.838(1.876) | 2.279(1.449) |
| Middle-high | Activities of Daily Living (ADL) | 4.899(2.344) | 7.867(4.173) | 10.322(3.247) | 7.396(2.766) | 6.912(2.74) |
| Middle-high | Pain | 1.438(1.113) | 5.471(3.548) | 4.405(5.004) | 2.456(1.372). | 1.438(1.113) |
| Middle-high | Chronic | -0.591(1.524) | -6.379(5.961) | -1.542(2.643) | -2.564(2.162) | -3.821(2.126). |
| Middle-high | Per household income | 499.141(1113.56) | -1076.527(2065.903) | -334.898(1574.562) | -155.639(1284.779) | -12.999(1262.713) |
| High | Gender | 0.599(1.045) | 2.535(1.885) | 1.507(1.43) | 1.022(1.222) | 0.673(1.162) |
| High | Education | 0.847(1.04) | 3.143(2.106) | 2.177(1.53) | 2.475(1.337). | 2.607(3.237) |
| High | Live alone | -4.261(2.373). | -22.275(22.474) | -3.394(2.797) | -3.862(2.555) | -3.439(2.252) |
| High | Activities of Daily Living (ADL) | 3.546(2.428) | 5.338(4.305) | 4.366(3.36) | 3.466(2.876) | 3.922(2.594) |
| High | Pain | 0.578(1.023) | -0.139(1.797) | -0.653(1.366) | -0.629(1.189) | 0.578(1.023) |
| High | Chronic | 0.623(1.426) | 5.317(2.931). | 2.343(1.955) | 0.465(1.53) | 0.623(1.426) |
| High | Per household income | -2807.797(6562.142) | 5075.866(11460.56) | -995.043(7555.292) | -6585.925(8200.14) | -6816.85(8226.134) |

† Data is present in effect value (sd), *** p < 0.001, ** p < 0.01, * p < 0.05, . p < 0.1.

‡ bw = IK means the bandwidth is the optimal bandwidth calculated by Imbens-Kalyanaraman method.

# Appendix 3-3 balance test for covariates (inpatient = yes) by different bandwidth and cutoff

| **Income group** | **Covariates** | **bw = no constrain** | **bw = 3** | **bw = 5** | **bw = 7** | **bw = IK** |
| --- | --- | --- | --- | --- | --- | --- |
| Total | Gender | 0.912(0.952) | -0.705(1.461) | -0.276(1.217) | 0.086(1.067) | -0.038(1.147) |
| Total | Education | 0.15(0.878) | 0.821(1.396) | -0.474(1.195) | -0.457(1.047) | 0.215(0.884) |
| Total | Live alone | -0.766(1.18) | 7.327(6.094) | 1.017(1.968) | 0.017(1.386) | -0.47(1.417) |
| Total | Activities of Daily Living (ADL) | 2.619(2.426) | 2.912(4.097) | 4.271(3.335) | 3.145(2.866) | 2.619(2.426) |
| Total | Pain | 1.182(0.869) | 1.954(1.45) | 1.596(1.174) | 1.04(1.007) | 1.182(0.869) |
| Total | Chronic | 0.127(1.027) | -1.4(1.562) | -1.318(1.361) | -1.176(1.239) | -0.877(1.189) |
| Total | Per household income | -962.708(1993.623) | -1101.076(2575.09) | -578.556(2374.269) | -491.269(2266.396) | 80.838(2348.603) |
| Low | Gender | 0.715(1.176) | -2.105(2.395) | -1.336(1.681) | -0.565(1.336) | 0.371(1.22) |
| Low | Education | 0.786(2.287) | -1.525(6.602) | -2.091(3.738) | -2.914(3.284) | -3.297(3.284) |
| Low | Live alone | -4.004(3.285) | -13.26(21.354) | -0.651(5.689) | -2.06(5.664) | 0.289(5.164) |
| Low | Activities of Daily Living (ADL) | -1.018(5.949) | 11.668(16.886) | 6.675(9.903) | 2.014(8.15) | -1.328(6.344) |
| Low | Pain | -8.553(4.758). | -39.789(97.063) | -3.858(7.453) | -2.631(4.753) | -1.89(4.821) |
| Low | Chronic | 0.197(2.6) | 2.985(6.239) | 3.229(3.741) | -0.329(3.193) | 0.197(2.6) |
| Low | Per household income | 178.827(200.979) | 939.721(650.443) | 507.313(340.086) | 168.054(262.05) | 235.683(211.342) |
| Low-middle | Gender | -0.011(1.823) | -5.253(7.165) | -0.384(2.35) | 0.364(2.232) | -1.15(1.969) |
| Low-middle | Education | 0.65(1.642) | 10.033(5.779). | 2.338(2.338) | 0.68(1.942) | 0.65(1.642) |
| Low-middle | Live alone | -0.325(2.658) | 5.545(7.747) | 1.096(4.971) | 3.347(4.817) | -0.325(2.658) |
| Low-middle | Activities of Daily Living (ADL) | 7.045(4.906) | 1.565(7.792) | 6.349(6.029) | 9.462(5.612) | 7.045(4.906) |
| Low-middle | Pain | 3.823(2.029). | 8.948(13.853) | 3.72(3.381) | 3.313(2.304) | 3.704(2.126). |
| Low-middle | Chronic | -1.382(2.459) | -3.619(5.591) | -13.789(16.384) | -4.139(4.234) | -1.382(2.459) |
| Low-middle | Per household income | -380.795(754.088) | -899.142(1423.16) | -502.102(1015.004) | -164.978(893.734) | -493.293(817.982) |
| Middle-high | Gender | 1.405(1.687) | -0.179(2.246) | -0.112(1.915) | 0.295(1.806) | 0.295(1.806) |
| Middle-high | Education | -0.263(1.581) | -0.591(2.181) | -1.494(2.015) | -1.15(1.822) | -0.263(1.581) |
| Middle-high | Live alone | 2.761(2.098) | 9.021(12.935) | 8.834(12.928) | 6.352(4.72) | 2.761(2.098) |
| Middle-high | Activities of Daily Living (ADL) | -1.551(3.646) | -1.929(5.207) | -0.799(4.413) | -3.748(4.119) | -2.486(3.916) |
| Middle-high | Pain | 0.928(1.611) | -0.207(2.133) | 1.075(1.95) | 0.25(1.771) | 0.187(1.775) |
| Middle-high | Chronic | 0.369(1.84) | -0.17(2.229) | 0.607(2.094) | -0.369(2.034) | -0.37(2.034) |
| Middle-high | Per household income | -2794.804(1445.644) | -3715.734(1908.839) | -3634.652(1692.364) | -3261.945(1621.315) | -3545.345(1577.511) |
| High | Gender | -1.711(2.622) | -5.523(19.446) | -1.917(6.572) | -2.043(3.754) | 0.696(3.083) |
| High | Education | -5.279(2.902). | -8.74(29.007) | -4.391(7.028) | -5.197(4.005) | -3.093(3.271) |
| High | Live alone | -3.261(3.373). | -10.887(8.626) | -4.633(3.633) | -3.597(2.39) | -3.439(2.252) |
| High | Activities of Daily Living (ADL) | 6.499(7.127) | -10.158(60.743) | -6.858(20.355) | 11.783(10.638) | 6.499(7.127) |
| High | Pain | 4.139(2.954) | -6.756(20.69) | -0.567(7.717) | 1.951(4.201) | 4.139(2.954) |
| High | Chronic | -2.101(4.24) | -2.546(2.04) | 0.523(1.551) | 0.564(1.5) | 0.623(1.426) |
| High | Per household income | 12295.969(8248.31) | 26766.895(48503.274) | -8515.63(18839.429) | 12086.93(11368.198) | 13825.878(11863.283) |

† Data is present in effect value (sd), *** p < 0.001, ** p < 0.01, * p < 0.05, . p < 0.1.
